# Alterations of the gut microbiota in patients with postherpetic neuralgia

**DOI:** 10.1186/s13568-023-01614-y

**Published:** 2023-10-06

**Authors:** Bo Jiao, Xueqin Cao, Caixia Zhang, Wencui Zhang, Shangchen Yu, Mi Zhang, Xianwei Zhang

**Affiliations:** 1 Department of Anesthesiology, Tongji Hospital of Tongji Medical College, Huazhong University of Science and Technology, Wuhan, Hubei Province China; 2 Department of Anesthesiology, Zhongnan Hospital, Wuhan University, Wuhan, Hubei Province China

**Keywords:** Postherpetic neuralgia, Neuropathic pain, Gut microbiota, 16S rRNA sequencing, Clinical symptoms

## Abstract

Postherpetic neuralgia (PHN) is a prevalent, intricate, and intractable form of neuropathic pain. The available evidence indicates that alterations in the gut microbiota are significant environmental determinants in the development of chronic neuropathic pain. Nevertheless, the correlation between the gut microbiota and PHN remains elusive. A cross-sectional study was performed on a cohort of 27 patients diagnosed with PHN and 27 matched healthy controls. Fecal samples were collected and subjected to microbiota analysis using 16S ribosomal RNA gene sequencing. Comparable levels of bacterial richness and diversity were observed in the gut microbiota of PHN patients and healthy controls. A significant difference was observed in 37 genera between the two groups. Furthermore, the LEfSe method revealed that the abundance levels of *Escherichia-Shigella*, *Streptococcus*, *Ligilactobacillus*, and *Clostridia_UCG-014_unclassified* were elevated in PHN patients, while *Eubacterium_hallii_group*, *Butyricicoccus*, *Tyzzerella*, *Dorea*, *Parasutterella*, *Romboutsia*, *Megamonas,* and *Agathobacter* genera were reduced in comparison to healthy controls. Significantly, the discriminant model utilizing the predominant microbiota exhibited efficacy in distinguishing PHN patients from healthy controls, with an area under the curve value of 0.824. Moreover, Spearman correlation analysis demonstrated noteworthy correlations between various gut microbiota and clinical symptoms, including disease course, anxiety state, sleep quality, heat pain, pain intensity, and itching intensity. Gut microbiota dysbiosis exists in PHN patients, microbiome differences could be used to distinguish PHN patients from normal healthy individuals with high sensitivity and specificity, and altered gut microbiota are related to clinical manifestations, suggesting potentially novel prevention and therapeutic directions of PHN.

**Supplementary Information:**

The online version contains supplementary material available at 10.1186/s13568-023-01614-y.

## Introduction

Postherpetic neuralgia (PHN) is a neuropathic pain that is commonly observed, and it endures for a duration of one month or more after the herpes zoster rash has resolved. PHN is the most frequent sequela of herpes zoster (Johnson and Rice 2014). It is clinically characterized by persistent pain, often accompanied by sensory anomalies, sleep disruption, and emotional comorbidities (Geha et al. 2007; Hunt and Mantyh 2001). The disease course of PHN can range from a few months to a lifetime, with an extremely difficult-to-treat nature and a significant impact on patients' quality of life. Despite extensive research, the pathogenesis of PHN remains incompletely understood. Besides neurological damage, risk factors may also act as disease triggers. Diverse clinical presentations and different severity of this disease indicate that risk factors, such as age, the number of herpes, pain intensity, and perceived mental stress may have vital roles in the pathogenesis of PHN (Jung et al. 2004; Takao et al. 2018; Yang et al. 2019). The gut microbiota has garnered significant attention as a potential environmental factor that may impact the health status of a host. There is mounting evidence linking the gut microbiota to various chronic pain conditions in humans, such as inflammatory pain and neuropathic pain (Alizadeh et al. 2022; Guo et al. 2019). However, it is currently unknown how the gut microbiota plays a role in PHN patients.

It was reported that the gut microbiota is involved in the functioning of pain-related receptors or ion channels and exerts direct control over both peripheral and central sensitization through the release of inflammatory mediators (Guo et al. 2019). Furthermore, mounting evidence supports the notion of a causal relationship between the gut microbiome and neuropathic pain in murine models, with potential mechanisms involving circulating bacterial metabolites and lipopolysaccharide levels, immune responses, and microglia activation (Minerbi and Shen 2022). Additionally, gut dysbiosis is linked to pain-associated behavior, including pain sensitivity and depression (Defaye et al. 2020; Lin et al. 2020). Convincing evidence suggests that intestinal microorganisms may play a crucial role in manifestations of neuropathic pain through gut-brain axes (Zhong et al. 2019). Whereas, no study has addressed the relevance between the gut microbiota and PHN to date, and correlations between the gut microbiota and clinical manifestations of PHN remain undefined.

Therefore, we hypothesized that PHN patients exhibit gut microbial dysbiosis that contributes to the development of PHN. To evaluate this hypothesis, we employed 16S rRNA sequencing to compare the gut microbiota composition of PHN patients and healthy controls, identified the biomarkers of PHN, and analyzed the relationship between microbiota and clinical manifestations of PHN. The present study will enhance the comprehension of gut microbiota in PHN pathogenesis, which may suggest new potential prevention strategies and therapeutic directions.

## Materials and methods

### Study population

This study was approved by the institutional ethics committee at Tongji Medical College, Huazhong University of Science and Technology (No.: S083), which was registered at the Chinese Clinical Trial Registry (No.: ChiCTR2100049883). In total, 54 participants were recruited. We conducted a cross-sectional study of 27 PHN patients and 27 matched healthy controls (HCs). All participants provided written informed consent. All participants recruited in our study resided in Hubei province for a long time to mitigate the potential impact of diverse lifestyles and regions on gut microbial compositions. Patients with PHN were obtained from the pain clinic of the Tongji Hospital, Tongji Medical College, Huazhong University of Science and Technology during their initial visit to the clinic.

Dietary data was obtained over a period of two weeks through the administration of questionnaires, as documented in Additional file 2: Table S1. Clinical data pertaining to each patient was obtained through the use of medical history records and interview-based questionnaires (Additional file 3: Table S2). The general characteristics, the psychological state, and sleep quality of all participants were also evaluated using questionnaires, as listed in Additional file 3: Table S2. Subsequent to the interview, fecal samples were collected from each participant between October 2021 and June 2022.

The inclusion criteria of the PHN group were as follows: (1) Over 18 years old; (2) Diagnosed with PHN; (3) Without serious complication. The HC group was recruited from healthy volunteers and was matched with the PHN group according to age, gender, and BMI. The following exclusion criteria were conducted for all groups: probiotics or prebiotics has been taken within one month; Hypertension; diabetes; obesity (body mass index (BMI) ≥ 30 kg/m); dyslipidemia; cancer; metabolic syndrome; a history of disease with an autoimmune component (such as rheumatoid arthritis); intestinal dysfunction (such as irritable bowel syndrome, Crohn’s disease, and inflammatory bowel disease); and abnormal liver and kidney function; medications such as pain medications, anti-inflammatory drugs, antibiotics, or psychotropic medications have been taken in the last 6 months.

### Clinical manifestations records and assessments

The disease course and the location of the lesion were recorded. Simultaneously, whether the following symptoms existed was also recorded, including preherpetic pain, spontaneous pain, dynamic mechanical allodynia, and sensory disturbance (itching or numbness).

The assessment of depression and anxiety states was conducted through the utilization of the Patient Health Questionnaire-9 (PHQ-9) and the Generalized Anxiety Disorder-7 (GAD-7), respectively (Kroenke et al. 2001; Spitzer et al. 2006). The scoring system for the PHQ-9 ranging from 0 to 4 indicate no depression, 5–9 indicate mild depression, 10–14 indicate moderate depression, 15–19 indicate moderately severe depression and ≥ 20 indicate severe depression (Wang et al. 2014). Likewise, scores of  ≥ 5, ≥ 10, ≥ 14, and ≥ 19 on the GAD-7 represent mild, moderate, moderately severe, and severe levels of anxiety (Spitzer et al. 2006). The sleep quality was assessed using the Insomnia Severity Index (ISI), with scores ranging from 0 to 28. According to the recommended score interpretation guidelines (Bastien et al. 2001), scores of 0–7 indicate no clinically significant insomnia, 8–14 indicate subthreshold insomnia, 15–21 indicate moderate clinical insomnia, and 22–28 indicate severe clinical insomnia. The cold pain and heat pain was assessed. The assessment of pain intensity was conducted through the utilization of a numerical rating scale (NRS), whereby a score of 0 indicated the absence of pain, while scores of 1–3, 4–6, and 7–10 represented mild, moderate, and severe pain, respectively. In a similar manner, the evaluation of itching intensity was performed using an NRS, with scores ranging from 0 to 10, where higher scores indicated a greater severity of itching intensity.

### Sample collection and sequencing

Following collection, fecal samples were immediately frozen at a temperature of −80 °C. Subsequently, fecal genomic DNA was extracted utilizing the CTAB method. The quality of the DNA extraction was assessed through agarose gel electrophoresis, and quantification was performed using an ultraviolet spectrophotometer. PCR amplification was carried out using primers 341F (5'-CCTACGGGNGGCWGCAG-3') and 805R(5'-GACTACHVGGGTATCTAATCC-3') which were specific to the V3–4 hypervariable regions of the 16S rRNA gene. The PCR products were purified using AMPure XT beads (Beckman Coulter Genomics, Danvers, MA, USA), quantified by Qubit (Invitrogen, USA), and recovery was facilitated using the AMPure XT beads recovery kit.

The purified PCR products were assessed using the Agilent 2100 Bioanalyzer (Agilent, USA) and Illumina’s library quantification kit (Kapa Biosciences, Woburn, MA, USA). A library concentration exceeding 2 nM was deemed acceptable. The qualified computer sequencing libraries, which featured non-repeatable index sequences, were subjected to gradient dilution, mixed in accordance with the required sequencing volume, and denatured into a single strand via NaOH for computer sequencing. The NovaSeq 6000 sequencer was employed for 2 × 250 bp double-ended sequencing, utilizing the NovaSeq 6000 SP Reagent Kit (500 cycles).

### 16S rRNA gene sequencing analysis

For the double-ended data obtained by sequencing, data separation of the sample was performed according to barcode information, and the connector and barcode sequence were removed. Primer sequence and balance base sequence of RawData were were removed according to the Cutadapt (V1.9.1). FLASH (v1.2.8, http://ccb.jhu.edu/software/FLASH/) was employed to concatenate each pair of paired-end reads into a longer tag based on the overlap area. Subsequently, Fqtrim (v0.94, http://ccb.jhu.edu/software/fqtrim/) was utilized to perform window quality scanning on the sequencing reads, with a default scanning window of 100 bp. If the average quality value in the window was lower than 20, the reading part from the beginning of the window to the end of 3’was truncated. Sequences whose length was less than 100 bp, or sequences with content of N (uncertain fuzzy bases) over 5% after truncation were removed. Additionally, the chimera sequence was eliminated through the utilization of Vsearch (v2.3.4, https://github.com/torognes/vsearch). Then, the high-quality Clean Data was finally obtained.

Divisive Amplicon Denoising Algorithm (DADA2) was invoked with QIIME 2 (Bolyen et al. 2019) denoise-paired for length filtering and denoising. The utilization of Amplicon Sequence Variants (ASVs) was employed for the creation of Operational Taxonomic Units (OTU) (Blaxter et al. 2005), resulting in the acquisition of the final ASV feature list and feature sequence.

Species annotation was conducted using the SILVA (Release 138, https://www.arbsilva.de/documentation/release138/) and NT-16S databases, based on the ASV sequence file, and the abundance statistics of each species in each sample were determined using the ASV abundance table. The confidence threshold for comments was set at 0.7.

### Statistical analyses

The statistical analysis was conducted utilizing SPSS 26.0 (SPSS Inc., Armonk, New York, United States). Continuous variables that displayed a normal distribution were expressed as mean ± standard deviation (SD), while non-normally distributed variables were presented as median (interquartile range, IQR). Percentages were used to represent other variables. Statistical significance was determined when p < 0.05.

The present study employed R software (Version 3.4.4) to conduct an analysis of alpha and beta diversity. Specifically, alpha diversity was assessed through the calculation of two indices, namely chao1 and shannon, using R's ggplot2 package. Meanwhile, beta diversity was utilized to examine the dissimilarities in gut microbial communities between PHN and HCs. To achieve this, a Principal Coordinate Analysis (PCoA) was performed, and the resulting multidimensional data were visualized using the ade4 and vegan packages in R software.

An analysis of differential abundance of intestinal flora was conducted at the class, order, family, and genus levels using the doBy package (Version 4.6.13) and ggplot2 package (Version 3.3.6) in R software (Version 4.1.3). Taxa with average abundance levels greater than 1%, P values less than 0.05, and Q values less than 0.05 were visualized (White et al. 2009). the linear discriminant analysis (LDA) effect size (LEfSe) method was utilized for biomarker discovery to identify the key fecal microbiota responsible for discriminating between the PHN group and the HC group. Sequentially, the Kruskal–Wallis rank sum test, Wilcoxon rank sum test, and LDA were executed to identify all distinctive biomarkers. The Receiver operating characteristic (ROC) analysis, a statistical tool utilized to evaluate the predictive accuracy of a model, was conducted utilizing SPSS 26.0, and the area under the curve (AUC) was employed to assess the ROC performance. The corrplot package in R software (Version 3.4.4) was employed to compute Spearman rank correlation between gut microbiota and clinical features.

## Results

### Basic information of recruited subjects

The general characteristics of all participants are summarized in Table 1. There were no statistically significant differences observed in age, gender, BMI, or education between the PHN and HC groups. However, the PHN group exhibited significantly more severe depressive symptoms (*p* = 0.002), anxiety symptoms (*p* = 0.006), and insomnia (*p* = 0.000) compared to the HC group. Additionally, the detailed clinical manifestations of the PHN group are shown in Table 2.

**Table 1:** Demographics assessments for all participants

	PHN (n = 27)	NC (n = 27)	P value
Age (y, mean ± SD)	58.93 ± 14.597	55.70 ± 15.384	0.433
Gender (M/F)	16/11	15/12	0.788
BMI (kg/m^2^, median ± SD)	21.65 ± 2.85	22.29 ± 2.33	0.37
Education (y, mean ± SD)	10.52 ± 3.23	10.41 ± 3.41	0.903
PHQ-9 (score, median [IQR])	8.0(3.0–13.0)	1.0 (0.0–6.0)	0.002
GAD-7 (score, median [IQR])	5.0(1.0- 7.0)	0.0 (0.0–4.0)	0.006
ISI (score, median [IQR])	10.0(8.0–13.0)	1.0 (1.0–7.0)	0.000

*SD* standard deviation, *BMI* body mass index, *n* sample size, *PHQ-9* Patient Health Questionnaire-9, *GAD-7* Generalized Anxiety Disorder Screener-7, *NRS* numerical rating scale, *ISI* Insomnia Severity Index

**Table 2:** Clinical manifestations of PHN

	PHN (n = 27)
Disease course (month), n (%)	
1–3	13 (48.15%)
4–6	4 (14.81%)
7–12	6 (22.22%)
> 12	4 (14.81%)
Location of lesion, n (%)	
Face (Trigeminal nerve region)	1 (3.70%)
Neck and upper limbs (Cervical nerve region)	8 (29.63%)
Trunk (Thoracic nerve region)	16 (59.26%)
Buttocks and lower limbs (Lumbar and Sacral nerves region)	2 (7.41%)
Preherpetic pain, n (%)	17 (62.96%)
Spontaneous pain, n (%)	25 (92.59%)
Dynamic mechanical allodynia, n (%)	16 (59.26%)
Sensory disturbance, n (%)	
Itching	15 (55.56%)
Numbness	13 (48.15%)
PHQ-9 (score), n (%)	
No depression (0–4)	10 (37.04%)
mild (5–9)	7 (25.93%)
moderate (10–14)	4 (14.81%)
moderate-severe (15–19)	4 (14.81%)
Severe (20–27)	2 (7.41%)
GAD-7 (score), n (%)	
No anxiety (0–4)	13 (48.15%)
Mild (5–9)	12 (44.44%)
Moderate (10–13)	0 (0%)
Moderate-severe (14–18)	0 (0%)
Severe (19–21)	2 (7.41%)
ISI (score), n (%)	
Insomnia without clinical significance (0–7)	6 (22.22%)
Subclinical insomnia (8–14)	16 (59.26%)
Clinical insomnia (moderate) (15–21)	4 (14.81%)
Clinical insomnia (severe) (22–28)	1 (3.70%)
Cold pain, n (%)	
Normal	10 (37.04%)
Hypalgesia	14 (51.85%)
Hyperpathia	3 (11.11%)
Heat pain, n (%)	
Normal	15 (55.56%)
Hypalgesia	8 (29.63%)
Hyperpathia	4 (14.81%)
Pain intensity (NRS 0–10), n (%)	
No pain (0)	1 (3.70%)
Mild (1–3)	12 (44.44%)
Moderate (4–6)	6 (22.22%)
Severe (7–10)	8 (29.63%)
Itching intensity (NRS 0–10), n (%)	
No itching (0)	9 (33.33%)
Mild (1–3)	12 (44.44%)
Moderate (4–6)	5 (18.52%)
Severe (7–10)	1 (3.70%)

*PHQ-9* Patient Health Questionnaire-9, *GAD-7* Generalized Anxiety Disorder Screener-7, *NRS* numerical rating scale, *ISI* Insomnia Severity Index

### Differences in gut microbiota composition between postherpetic neuralgia patients and healthy controls

**Fig. 1:**
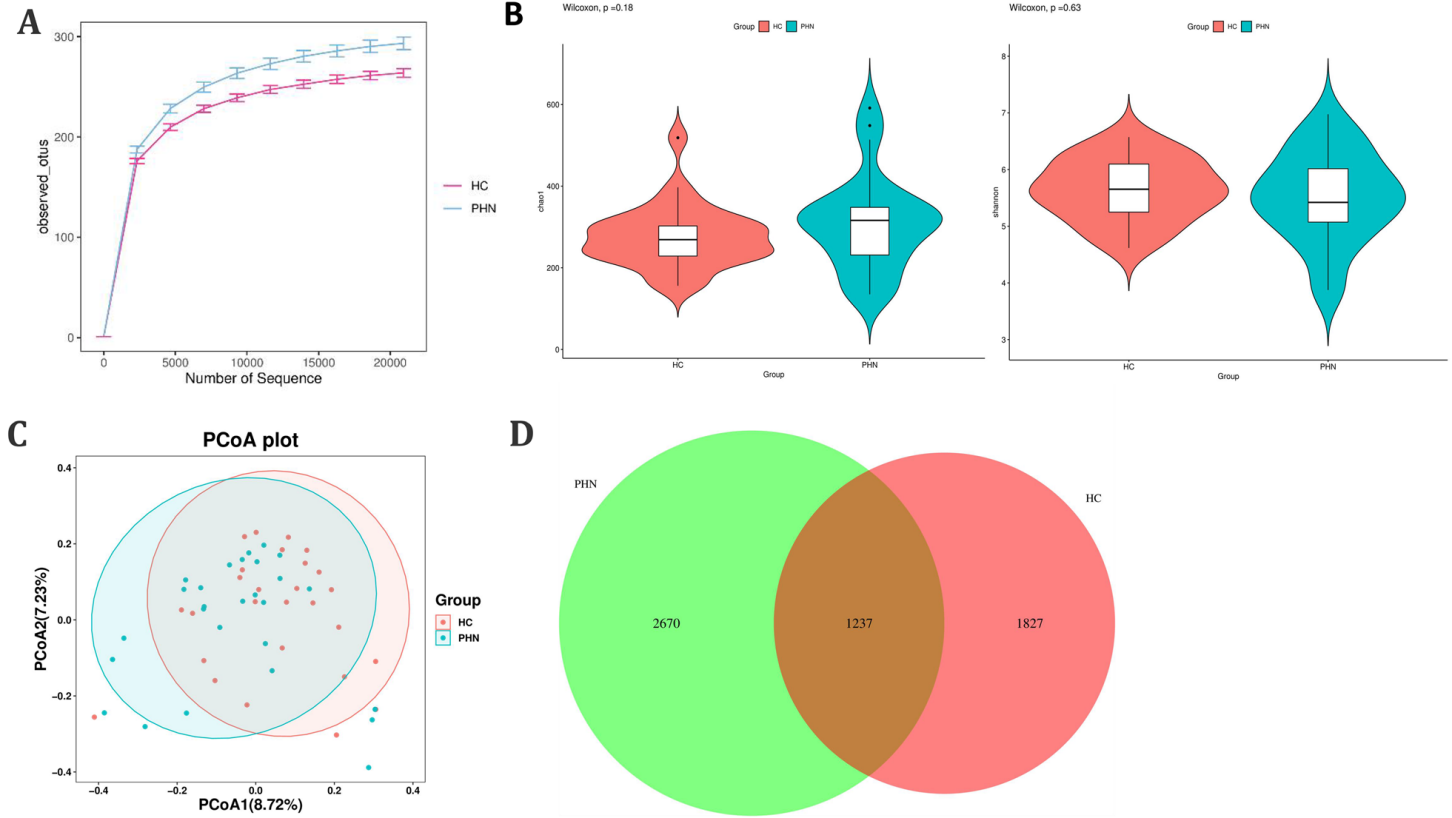
Rarefaction curves and comparison of diversity indexes between PHN patients and HCs. **A** Rarefaction curves of patients with PHN and HCs. **B** α-diversity indexes in PHN patients and HCs (chao1, Shannon). **C** PcoA for β-diversity analysis. **D** Venn diagram

The examination of rarefaction curves at the species level indicates that the sequencing sample size employed in this study was of sufficient magnitude and reliability. Furthermore, the gut microbiota in PHN patients demonstrated a tendency towards greater species richness (observed OTU number) in comparison to that of the healthy controls (Fig. 1A). The analysis of α-diversity involved the consideration of both species richness and evenness, as measured by the Chao1 index and the Shannon diversity index, respectively. However, no statistically significant differences were observed in the comparison of α-diversity between the two groups (*p* > 0.05) (Fig. 1B). The PCoA plot, as determined by the β-diversity analysis, indicated no statistically significant differences in the degree of similarity among microbial communities between the two groups (Fig. 1C). Additionally, the Venn diagram analysis revealed that 1237 ASVs were common to both the PHN and HC groups, while 2670 and 1827 ASVs were unique to PHN patients and HCs, respectively (Fig. 1D).

**Fig. 2:**
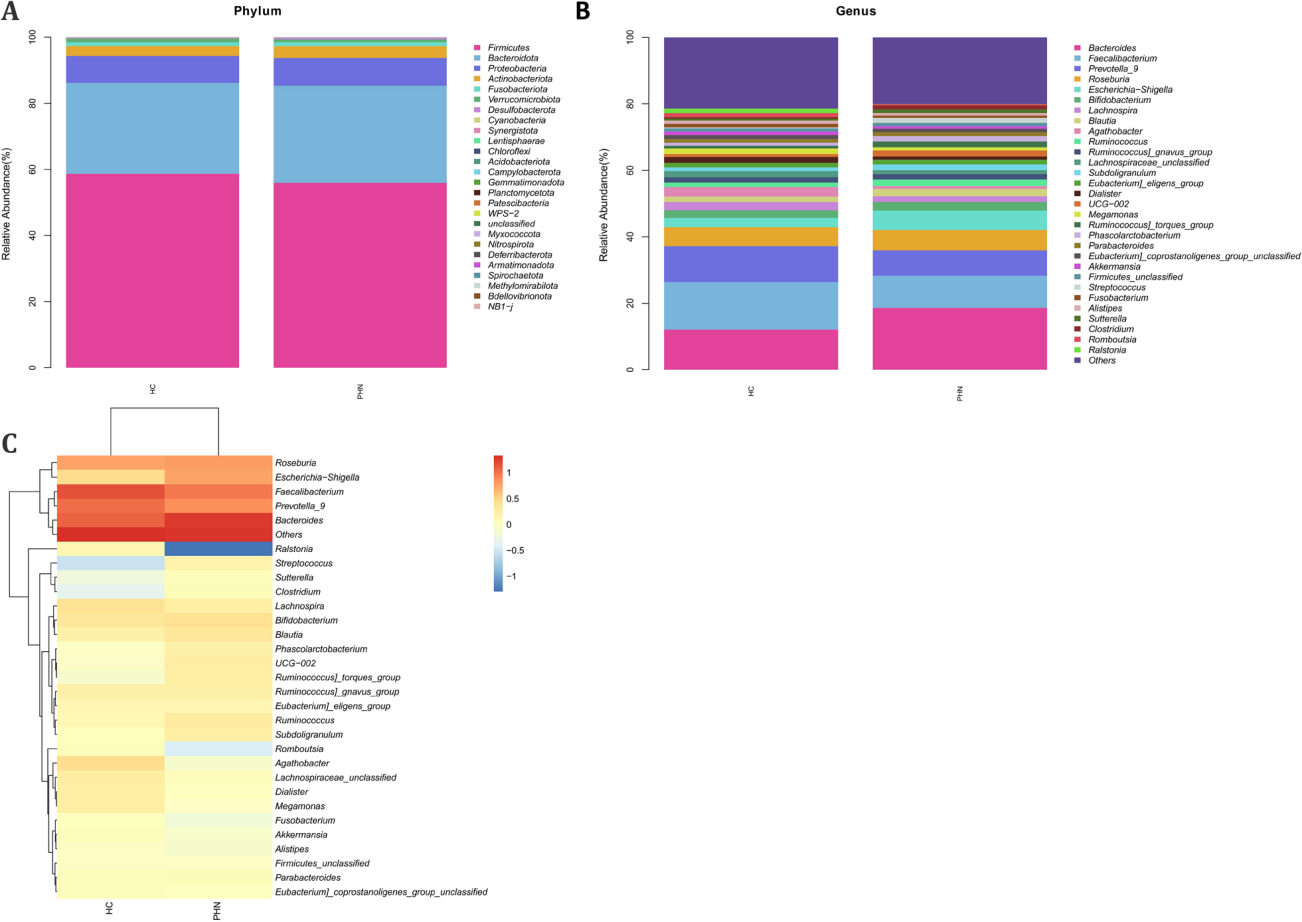
Community Profiling analysis showing differential relative abundances of fecal microbiota in PHN patients and HCs. **A** Microbiome composition of the two groups at the phylum level. **B** Microbiome composition of the two groups at the genus level. **C** Relative abundance of the top 30 genus in each sample

The analysis of Community Profiling revealed no statistically significant differences between the PHN group and HCs from phylum to species levels (Additional file 1: Figure S1 and Fig. 2). The bacterial phyla *Firmicutes*, *Bacteroidota*, and *Proteobacteria* were found to be predominant (Fig. 2A). In comparison to HCs, PHN patients exhibited lower levels of *Firmicutes*, but higher levels of *Bacteroidota* and *Proteobacteria*. At the class level, *Bacteroidota* and *Gammaproteobacteria* were found to be more abundant in PHN patients, while *Clostridia* and *Negativicutes* were more prevalent in HCs. At the level of order, it was observed that PHN patients exhibited an increase in *Bacteroidales* and *Enterobacterales*, but a decrease in *Oscillospirales* and *Lachnospirales*, in comparison to HCs. The family level analysis revealed that *Bacteroidaceae* and *Enterobacteriaceae* were more abundant in the PHN group, while *Lachnospiraceae*, *Ruminococcaceae*, and *Prevotellaceae* were more prevalent in the healthy group. At the genus level, *Bacteroides* and *Faecalibacterium* were the most dominant in both groups, with *Bacteroides* being more abundant in the PHN group, and *Faecalibacterium* and *Prevotella_9* being more prevalent in the HCs group (Fig. 2B and C). At the species level, the abundance of *Roseburia_unclassified* and *Escherichia-Shigella_unclassified* was observed to be higher in the PHN group, whereas *Faecalibacterium_unclassified* and *Prevotella_9_unclassified* were more prevalent in the HCs.

These findings suggest that the gut microbiota of PHN patients and healthy controls exhibit comparable levels of bacterial richness and diversity, but the overall composition of the gut microbiota differs significantly between the two groups.

### Alteration in gut microbiota between postherpetic neuralgia patients and healthy controls

**Fig. 3:**
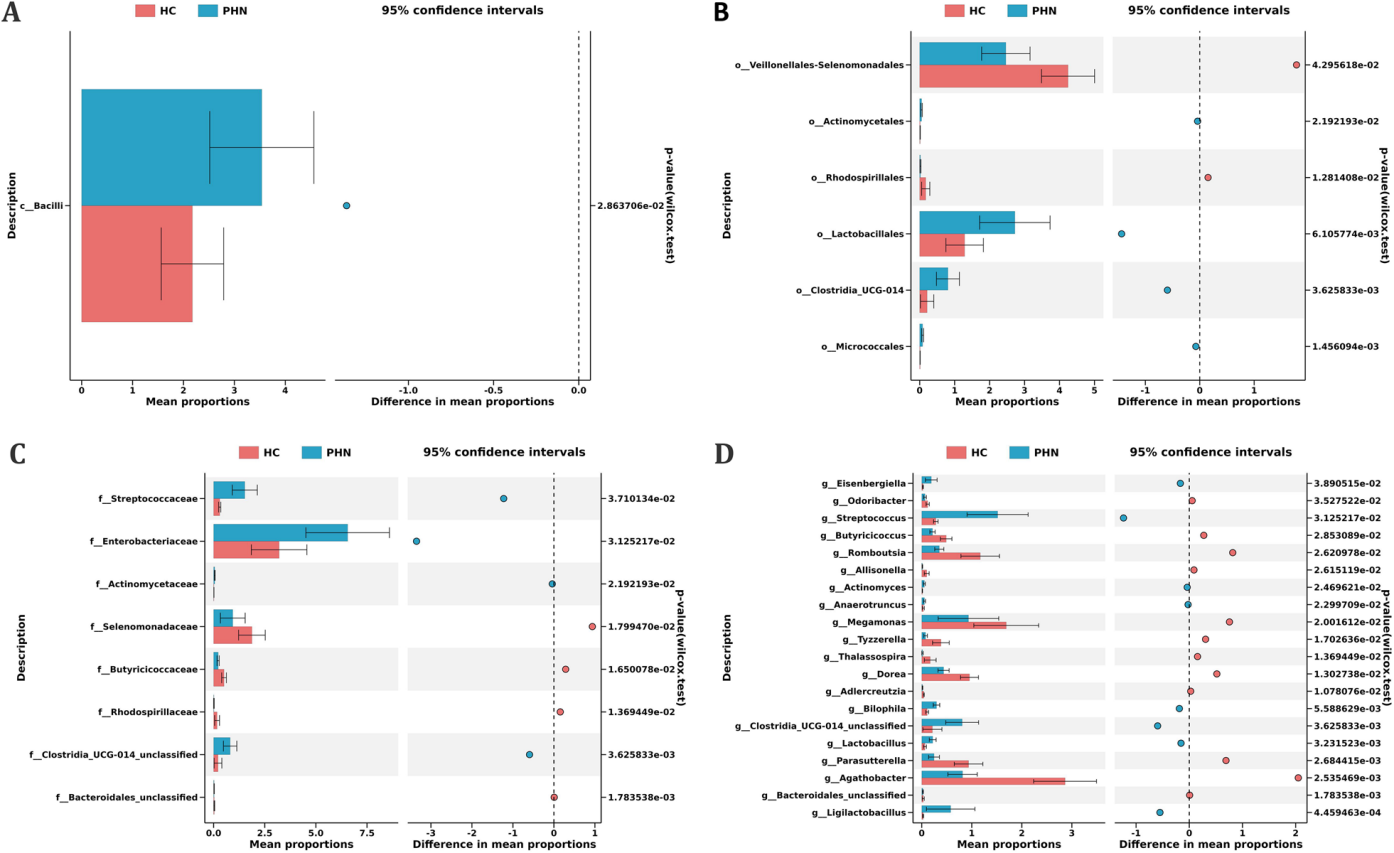
Gut microbiota differences between PHN patients and controls. Gut microbiota is compared between PHN patients and healthy control subjects at class (**A**), order (**B**), family (**C**), and genus (**D**) levels. Only the taxa with average abundance levels exceeding 1% are plotted. The bars on the left side of each figure show the relative abundance. On the right side of each figure, the center of circles represents the difference between the means of the two groups. The error bars represent the 95% confidence interval. *P* values of unpaired t-test are listed on the right

A significant difference was observed between PHN patients and HCs in 3 classes, 13 orders, 16 families, 37 genera, and 46 species of gut microbiota (Additional file 4: Table S3). Taxa with average abundance levels exceeding 1% were graphically represented. Specifically, at the class level, the proportion of *Bacilli* was found to be higher in the PHN group compared to the HCs (Fig. 3A). At the order level, *Actinomycetales*, *Lactobacillales*, *Clostridia_UCG-014*, and *Micrococcales* were more abundant in the PHN group, while *Veillonellales-Selenomonadales*, and *Rhodospirillales* were more prevalent in the HCs (Fig. 3B). At the family level, proportions of *Streptococcaceae*, *Enterobacteriaceae*, *Actinomycetaceae*, and *Clostridia_UCG-014 unclassified* were higher in the PHN group than that in the HCs, while *Selenomonadaceae*, *Butyricicoccaceae*, *Rhodospirillaceae*, and *Bacteroidales_unclassified* were lower (Fig. 3C). At the genus level, 20 genera displayed substantial variation between the PHN group and HCs. Specifically, the proportions of *Odoribacter*, *Butyricicoccus*, *Romboutsia*, *Allisonella*, *Megamonas*, *Tyzzerella*, *Thalassospira*, *Dorea*, *Adlercreutzia*, *Parasutterella*, *Agathobacter*, and *Bacteroidales_unclassified* genera were decreased, whereas the proportions of *Eisenbergiella*, *Streptococcus*, *Actinomyces*, *Anaerotruncus*, *Bilophila*, *Clostridia_UCG-014_unclassified*, *Lactobacillus*, and *Ligilactobacillus* genera were increased in PHN patients (Fig. 3D).

**Fig. 4:**
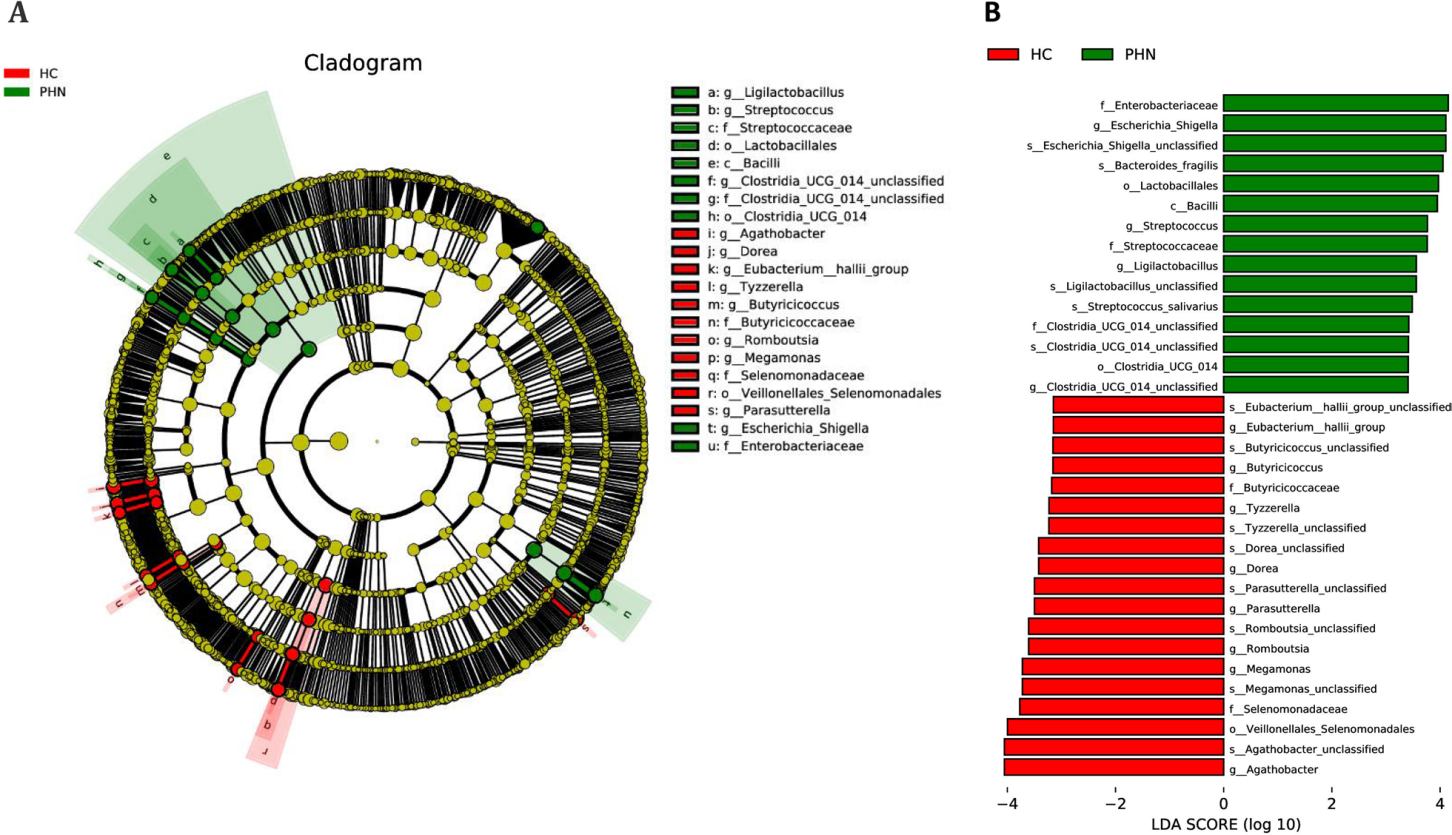
LEfSe identified the taxa with the greatest differences in abundance between PHN patients and HCs. **A** Cladogram showing differential bacterial abundance in the PHN and control groups. **B** Microbiome biomarkers were identified. The green color represents the PHN group, and the red color represents the HCs. LDA score for discriminative features > 3.0

To further explore the specific bacterial taxa related to PHN, the LEfSe algorithm was employed to assess the abundance of the fecal microbiota (Fig. 4). A cladogram was generated to facilitate the comparison of the phylogenetic distribution between healthy controls and PHN patients. The results revealed significant differences at each taxonomic level analyzed (LDA > 3, *p* < 0.05), with 34 differential ASVs being identified. Concretely, one class, two orders, three families, four genera, and five species were found to be enriched in PHN patients, while one order, two families, eight genera, and eight species were more abundant in HCs (*p* < 0.05). In comparison to NCs, patients with PHN exhibited ten enriched ASVs primarily belonging to the class *Bacilli*. Notably, the families *Streptococcaceae*, *Clostridia_UCG-014_unclassified*, and *Enterobacteriaceae* were more abundant in PHN patients, while the *Butyricicoccaceae* and *Selenomonadaceae* families were more prevalent in NCs. At the genus level, *Escherichia-Shigella*, *Streptococcus*, *Ligilactobacillus*, and *Clostridia_UCG-014_unclassified* were enriched in PHN patients, whereas *Eubacterium_hallii_group*, *Butyricicoccus*, *Tyzzerella*, *Dorea*, *Parasutterella*, *Romboutsia*, *Megamonas,* and *Agathobacter* were enriched in NCs. The findings indicate notable variations in the intestinal microbiota between PHN and HC groups.

### ROC curve analysis

**Fig. 5:**
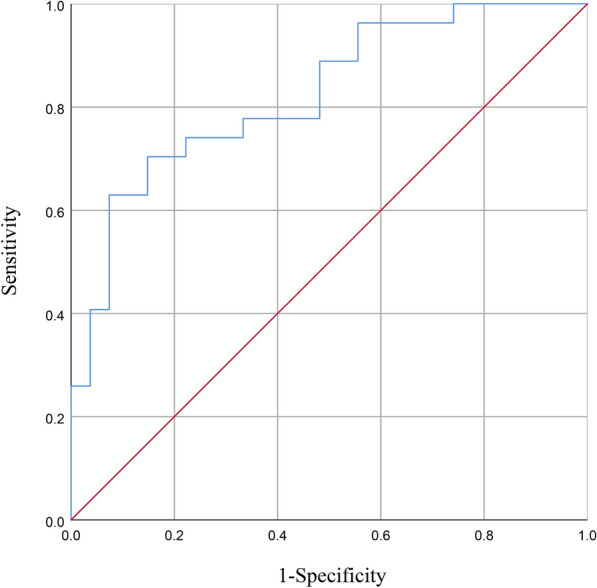
ROC curves of the gut genus bacterium relative abundance for the prediction of PHN. Vertical coordinate indicated the sensitivity of prediction, horizontal coordinate indicated the 1-specificity of prediction, AUC > 0.5 indicated a predictive efficiency of the gut bacterium

Consequently, our objective was to ascertain potential biomarkers that could differentiate between the two groups. We identified the five most prominent genera (*Escherichia_Shigella*, *Agathobacter*, *Streptococcus*, *Megamonas*, and *Romboutsia*) based on their LDA value, and subsequently employed these microbiota to construct the ROC curve (Fig. 5). The AUC of the ROC curve was determined to be 0.824, indicating that the model possessed the ability to differentiate between the two groups. Additionally, the model exhibited a specificity and sensitivity of 92.6% and 63.0%, respectively, signifying a notable diagnostic efficacy. These findings suggest that the gut microbiota may serve as a robust predictor of PHN.

### Clinical manifestations correlated with the gut microbiota

**Fig. 6:**
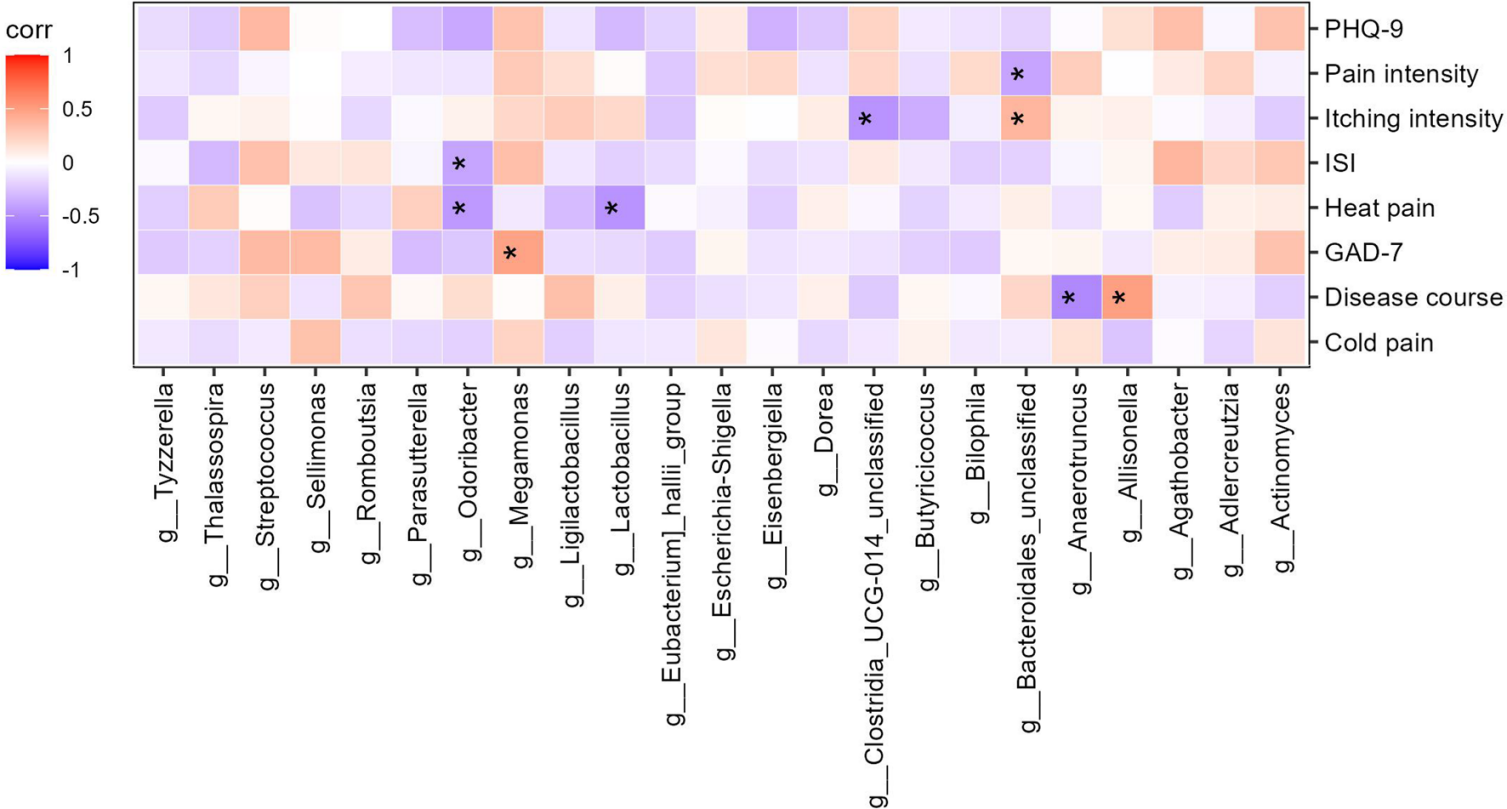
Spearman correlation analysis of PHN clinical symptoms. The intensity of the color indicates the r value (correlation). The red color represents a positive score, and the purple color represents a negative one. *p < 0.05

The associations between the gut microbiota and clinical manifestations were investigated (Fig. 6). The results revealed a negative correlation between *Anaerotruncus* and disease course, while *Allisonella* exhibited a positive correlation with disease course. Additionally, a positive trend was observed between *Megamonas* abundance and GAD-7, and *Odoribacter* demonstrated a direct negative association with ISI. Moreover, *Lactobacillus* and *Odoribacter* were negatively correlated with heat pain. *Bacteroidales_unclassified* was negatively associated with pain intensity, but positively correlated with itching intensity. Additionally, Clostridia_UCG-014_unclassified was negatively associated with itching intensity.

## Discussion

In recent years, accumulating evidence has demonstrated that the disorder of gut microbiota plays a significant role in modulating the development of neuropathic pain (Lin et al. 2020). This study employed 16S rRNA sequencing analysis to characterize the gut microbiota of PHN patients for the first time. The results revealed that PHN patients displayed an altered gut microbiota composition. Furthermore, a prediction model was developed based on the results of LEfSe and achieved high values of AUC. Moreover, the Spearman correlation analysis revealed robust associations between differentiated gut microbiota and diverse clinical presentations. These results indicate that dysbiosis of gut microbiota is a crucial factor in the pathogenesis of PHN.

In our study, we observed a lower abundance of *Firmicutes* at the phylum level in PHN patients, while *Bacteroidota* exhibited a higher abundance. Several previous studies also observed marked decreases in *Firmicutes* and increase in *Bacteroidota* in the intestines of patients with neuralgia (Lin et al. 2020; Zhang et al. 2019). It is noteworthy that phylum *Firmicutes* is known to play a crucial role in regulating inflammatory responses and human metabolic functions (Bhat and Kapila 2017). The depletion of the *Firmicutes* phylum may result in the production of pro-inflammatory cytokines and toxic metabolites, while simultaneously reducing the presence of beneficial substances such as short-chain fatty acids (SCFAs), ultimately leading to damage to the gut epithelial barrier. The *Bacteroidota* phylum is characterized by its major outer membrane component, lipopolysaccharide (LPS). LPS is known to play a critical role in the initiation of systemic inflammation and the release of pro-inflammatory cytokines. Thus, the missing phylum *Firmicutes* and enriched phylum *Bacteroidota* in PHN patients may exacerbate neuroinflammation in humans, thereby augmenting the likelihood and advancement of PHN.

At the genus level, a reduction in the abundance levels of many microbiotas was observed in PHN patients. According to the LEfSe results, *Eubacterium_hallii_group*, *Butyricicoccus*, *Tyzzerella*, *Dorea*, *Parasutterella*, *Romboutsia*, *Megamonas*, and *Agathobacter* were decreased in PHN patients. Previous research has established the significant roles played by these species in preserving human health. *Eubacterium_hallii*, *Butyricicoccus*, and *Agathobacter* were shown to produce butyrate (Duncan et al. 2004; Geirnaert et al. 2014; Iversen et al. 2022). According to Hamer et al., butyrate serves as the primary energy source for colonocytes, thereby promoting the maintenance of gastrointestinal health by enhancing epithelial barrier integrity and suppressing inflammation (Hamer et al. 2008). Furthermore, recent studies have shown that butyrate possesses the ability to ameliorate neuropathic pain (Bonomo et al. 2020; Kukkar et al. 2014). The genera *Tyzzerella, Dorea*, *Parasutterella*, *Romboutsia*, and *Megamonas* have been shown to produce SCFAs, which are important for maintaining the health of the gut lining and supporting immune function (Huang et al. 2022; Jiao et al. 2018; Xiao et al. 2020; Xu et al. 2021; Zhang et al. 2022). Therefore, we hypothesized that a decrease in these genera of bacteria might be detrimental to PHN by affecting the abundance of SCFAs. Furthermore, *Dorea* has been demonstrated negatively linked to inflammatory diseases (Bajaj et al. 2012). It was shown that *Romboutsia* and *Megamonas* may be beneficial for individuals with inflammatory bowel disease or other inflammatory conditions because of their anti-inflammatory effects (Qiu et al. 2020; Yu et al. 2021). Consequently, the reduced levels of these species in PHN patients may contribute to the destruction of the intestinal mucosal barrier, thereby triggering inflammation and immune responses and ultimately exacerbating the pathology of PHN.

While, the abundance levels of several genera were found to be increased in PHN patients according to the LEfSe results. Specifically, *Escherichia_Shigella*, *Streptococcus*, *Ligilactobacillus*, and *Clostridia_UCG-014_unclassified* were observed to be elevated in PHN patients. Previous studies have linked certain species with increased abundance levels to inflammatory diseases or neuropathic pain. Notably, *Escherichia_Shigella* has been associated with a pro-inflammatory status, and chronic and persistent peripheral inflammation has been observed in individuals with persistent infection with this species (Qiu et al. 2020). Similar to our findings, previous studies also revealed remarkably increased *Streptococcus* in rats with CCI-induced neuropathic pain (Chen et al. 2021). Additionally, *Streptococcus* is associated with inflammatory pain (Chakravarthy et al. 2014; Guo et al. 2019). Thus, we hypothesized that an increase in these genera of bacteria might contribute to PHN development by exacerbating inflammation. Interestingly, it was reported that *Ligilactobacillus* and *Clostridia_UCG-014_unclassified* may be involved in maintaining a healthy gut environment (Guerrero Sanchez et al. 2022; Liu et al. 2021). It is noteworthy that in our study, there was a negative correlation between *Clostridia_UCG-014_unclassified* and itching intensity in patients with PHN. Further investigation is necessary to determine the potential impact of elevated levels of these particular species on PHN in humans.

We used LEfSe analysis to select five species based on their LDA values for use as biomarkers for predicting disease status. A ROC Curve was constructed using the abundance of the top five genera, selected from a pool of 12 genera, resulting in an AUC value of 0.824. Therefore, it was confirmed that the discriminant model could effectively distinguish PHN patients from healthy controls, which suggests that the gut microbiota could be used to forecast PHN. Additionally, a heat map was employed to depict the associations between species and clinical phenotype, revealing that certain gut microbiota exhibited significant correlations with clinical manifestations related to PHN, including disease course, anxiety states, sleep quality, heat pain, pain intensity, and itching intensity. These outcomes establish a basis for investigating the interplay between the gut microbiota and the host in relation to the onset of PHN.

One of the primary strengths of our study is the utilization of the 16S rRNA gene sequencing method to profile the gut microbiota of patients with PHN. Additionally, we have developed a pioneering predictive model and conducted Spearman correlation analysis to explore associations between differentiated gut microbiota and clinical presentations in PHN. Theoretically, the variability of the microbiome may offer a degree of plausible explanations for longstanding clinical enigmas, such as the development of chronic pain in certain patients following the resolution of herpes zoster rash and the variability of clinical symptoms among patients. However, it is imperative to acknowledge that this study is in its preliminary stages and is subject to several limitations that require further investigation. Firstly, our study is a single-center, cross-sectional study with a limited sample size. Secondly, despite age-, gender-, BMI-, and diet matching of PHN patients in the analysis, other confounding factors such as stress may have influenced our results. Thirdly, it was suggested that SCFAs may be implicated in the development of PHN in our study, while the association of fecal or plasma levels of SCFAs with gut microbiota in PHN patients was not further explored. Additionally, the impact of certain treatments for PHN, including pharmacological, nonpharmacological, and interventional, on the gut microbiota remains unclear. Lastly, our research lacks animal experiments to investigate the underlying mechanisms linking gut microbiota dysbiosis to the development of PHN, as well as animal experiments exploring the potential of fecal microbiota transplantation as a treatment for PHN. Consequently, further research is imperative, incorporating larger sample sizes, multicenter designs, follow-up studies after treatment, animal experimentation, and innovative research techniques such as metagenomics and metabonomics. This will facilitate the exploration of potential causal mechanisms between gut microbiota and PHN, thereby providing guidance for future scientific investigations and interventions aimed at the prevention and treatment of PHN.

In summary, our study revealed a significant correlation between gut microbiomes and PHN, suggesting that disruptions in gut microbiota may play a role in the development of PHN. Our results provide evidence to support the potential use of gut microbiota as a predictive tool for PHN and targeting gut microbiota as a novel therapeutic approach for PHN.

### Supplementary Information

**Additional file 1: Figure S1.** The bacterial community in both groups at diferent taxonomic levels. Bar graphs indicated the relative abundance of class-level, order-level, family-level taxa, and species-level.

**Additional file 2: Table S1.** Dietary data of all participants.

**Additional file 3: Table S2.** Clinical information for all samples.

**Additional file 4: Table S3.** The difference of gut microbiota abundance between PHN patients and HCs.

## Data Availability

The data that support the findings of this study are openly available in BioProject at https://www.ncbi.nlm.nih.gov/bioproject/PRJNA975052, reference number PRJNA975052.
