# Supplementary figures and images for: Alterations of the gut microbiota in patients with postherpetic neuralgia
Source: AMB Express. 2023 Oct 6;13:108. doi: 10.1186/s13568-023-01614-y (PMC10558420; doi:10.1186/s13568-023-01614-y)

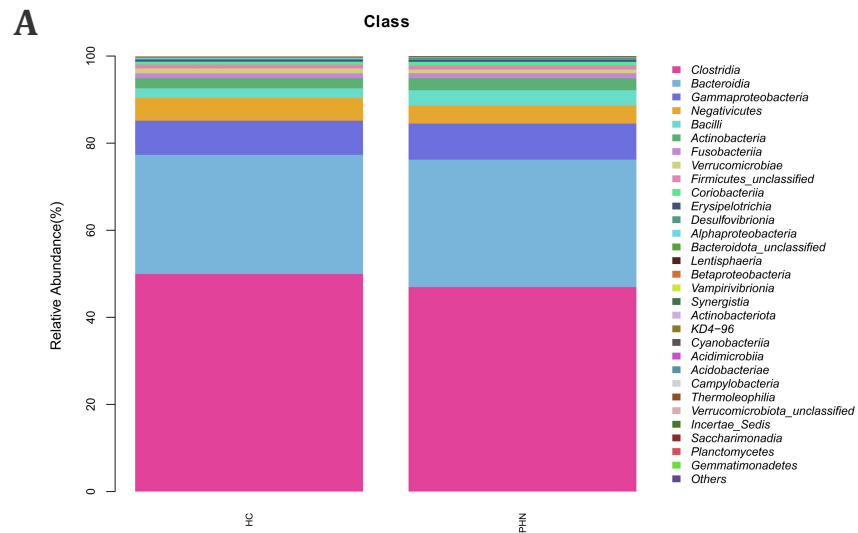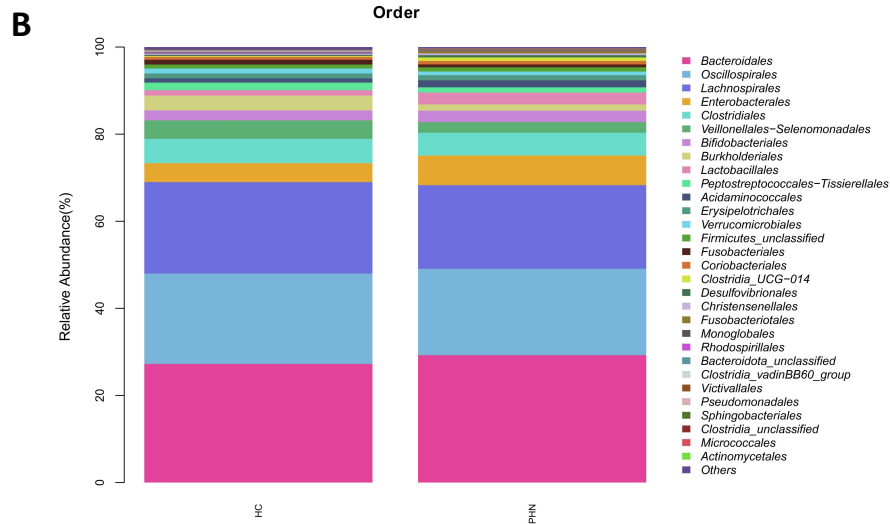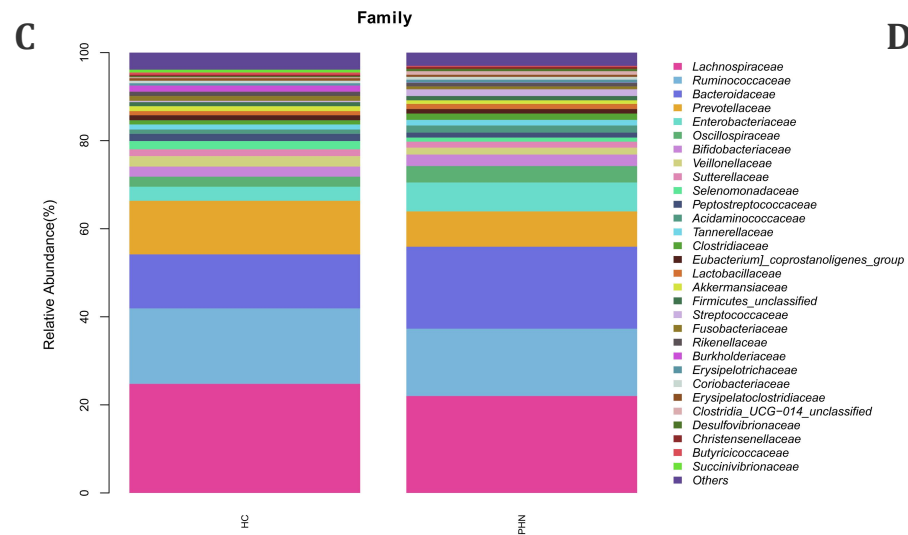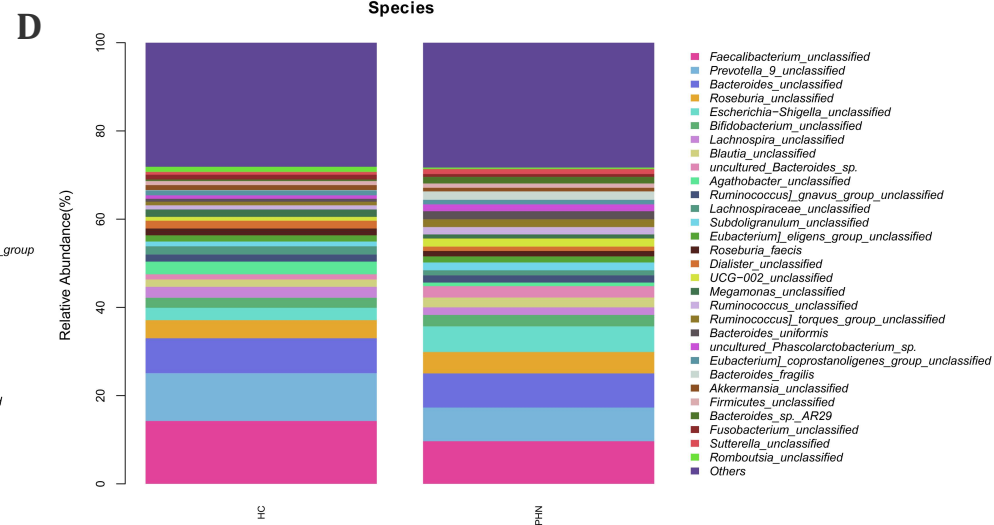

Supplement: Supplementary file 1 — Additional file 1: Figure S1. The bacterial community in both groups at diferent taxonomic levels. Bar graphs indicated the relative abundance of class-level, order-level, family-level taxa, and species-level. [file 13568_2023_1614_MOESM1_ESM.pdf]
